# Overcoming the Speed Limit of Four-Way DNA Branch Migration with Bulges in Toeholds

**DOI:** 10.1021/acs.nanolett.5c03063

**Published:** 2025-09-04

**Authors:** Samia Bakhtawar, Francesca Smith, Aditya Sengar, Guy-Bart V. Stan, John Goertz, Molly Stevens, Thomas E. Ouldridge, Wooli Bae

**Affiliations:** ‡ School of Mathematics and Physics, Faculty of Engineering and Physical Sciences, 3660 University of Surrey, Guildford GU2 7XH, United Kingdom; § Imperial College Centre for Synthetic Biology and Department of Bioengineering, Imperial College London, South Kensington Campus, London SW7 2AZ, United Kingdom; ∥ Department of Materials, Department of Bioengineering and Institute of Biomedical Engineering, 4615 Imperial College London, London SW7 2AZ, United Kingdom; ⊥ Department of Physiology, Anatomy and Genetics, Department of Engineering Science, Kavli Institute for Nanoscience Discovery, 6396 University of Oxford, Oxford OX1 3QU, United Kingdom

**Keywords:** toehold-mediated strand displacement (TMSD), four-way DNA branch migration, Holliday junction, oxDNA

## Abstract

Dynamic DNA nanotechnology creates programmable reaction networks and nanodevices by using DNA strands. The key reaction in dynamic DNA nanotechnology is the exchange of DNA strands between different molecular species, achieved through three- and four-way strand exchange reactions. While both reactions have been widely used, the four-way exchange reaction has traditionally been slower and less efficient than the three-way reaction. In this paper, we describe a new mechanism to optimize the kinetics of the four-way strand exchange reaction by adding bulges to the toeholds of the four-way DNA complexes involved in the reaction. These bulges facilitate an alternative branch migration mechanism and destabilize the four-way DNA junction, increasing the four-way strand exchange rate by an order of magnitude. This advancement has the potential to expand the field of dynamic DNA nanotechnology by enabling efficient four-way strand exchange reactions for *in vivo* applications.

## Graphical abstract

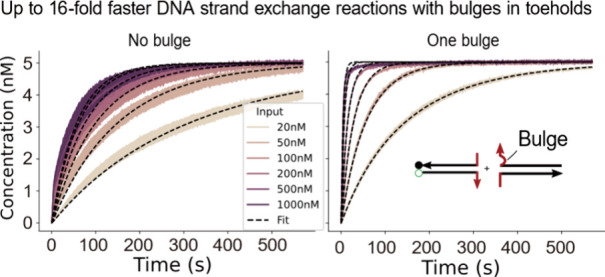

In the field of DNA nanotechnology, DNA is utilized as a programmable building block to build nanostructures and synthetic reaction circuits.^1^ These circuits deploy reactions that are programmed via Watson–Crick complementarity. The resultant field of dynamic DNA nanotechnology has applications, including dynamic nanomachines,^2–10^ sensing devices,^11,12^ and reaction networks for synthetic biology and molecular computing.^13–17^

Toehold-mediated strand displacement (TMSD), in which DNA and RNA strands are exchanged in a highly specific manner with controllable kinetics,^18,19^ is the fundamental reaction in dynamic DNA nanotechnology. A typical TMSD reaction involves the exchange of one strand for another within a complex via a three-way DNA branch migration (Figure 1a). In this reaction, a single-stranded invader hybridizes to a substrate–incumbent complex via a short single-stranded overhang, known as the toehold. Following toehold binding, a three-way junction forms between the invader, substrate, and incumbent strands, which can migrate back and forth in a random walk fashion until the invader either completely detaches or displaces the incumbent strand.

**Figure 1:**
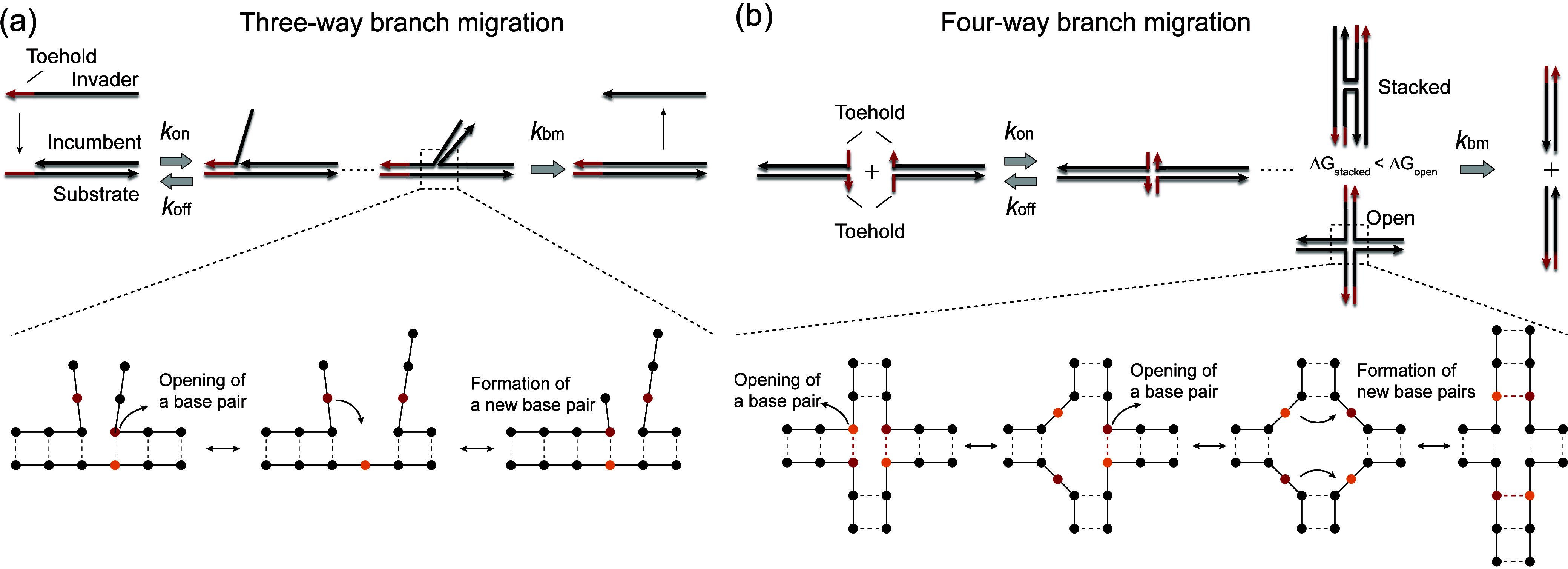
Comparison of three- and four-way branch migration mechanisms. (a) In three-way branch migration, a single-stranded invader hybridizes to an incumbent–substrate complex via the toehold before progressively displacing the incumbent strand (upper scheme). During the displacement, branch migration is thought to occur by stochastic opening of an incumbent–substrate base pair and its replacement by an invader–substrate base pair (lower scheme). (b) In four-way branch migration, two duplexes hybridize via two toehold sequences, forming a Holliday junction (upper scheme). Here, branch migration involves the exchange of two base pairs (lower scheme).

Notably, while three-way branch migrations are more frequently employed in dynamic nucleic acid reaction circuits, one can also use a four-way branch migration mechanism in dynamic molecular reactions.^20–22^ Four-way branch migration requires the formation of a Holliday junction between two DNA duplexes via hybridization of two toehold sequences (Figure 1b). The Holliday junction is an important structure that appears during *in vivo* homologous recombination,^23^ which is foundational to DNA double-strand break repair^24^ and DNA replication.^25^ The crystal structure of the Holliday junction revealed that the four arms of the Holliday junctions form a stable stacked monomer in the presence of a high salt concentration (Figure 1b, stacked form) with the occasional transition to an open, unstacked state.^26,27^ Branch migration can proceed by the simultaneous exchange of two base pairs at the junction, until either complete dissociation into the original duplexes or exchange of strands has occurred.^23^

The four-way branch migration scheme offers several advantages over three-way branch migration, and it still allows for the design of complex reaction circuits, despite requiring more species to execute logic.^22^ Four-way branch migration eliminates the need for single-stranded DNA species, thereby slowing down the degradation^28^ and minimizing undesired crosstalk with endogenous RNAs for *in vivo* applications.^29^

A fundamental strength of three-way strand displacement lies in its fast and highly tunable kinetics. This process is typically modeled as a two-step process involving three rate constants: *k*_on_, *k*_off_, and *k*_bm_. These represent the rate constants of bimolecular binding through the toehold, toehold unbinding, and branch migration, respectively (Figure 1a and b).^18,19^ In a typical three-way strand displacement reaction, the toehold length is crucial in controlling the kinetics as it heavily influences *k*_off_. However, when using longer toeholds and high concentrations where binding is saturated, *k*_bm_ sets the speed limit of the reaction and it is generally high.^18,19^

While four-way strand exchange can be modeled and tuned with the same set of parameters and toehold lengths as three-way strand displacement, its *k*_bm_ is fundamentally much slower, 2–3 orders of magnitude lower.^19,23^ This significantly limits the maximum reaction speed regardless of the toehold length or reactant concentration.^23^ This difference in speed comes from their molecular mechanisms. Three-way branch migration requires the spontaneous opening of a single base pair, followed by the formation of a new base pair for each step (Figure 1a). In contrast, four-way branch migration requires simultaneous opening of two base pairs, followed by the subsequent formation of two new base pairs to complete a branch migration step at the junction (Figure 1b). This process is further hindered by the increased stabilization of stacked forms of a Holliday junction at high salt concentrations (Figure 1b).^27,30^

Our study addresses this inherent kinetic limitation by introducing bulges at the junction to accelerate branch migration, thereby significantly enhancing the reaction rate by 1 order of magnitude and expanding the practical utility of four-way branch migration-based systems. We use oxDNA simulation^31^ to explain aspects of the system performance. Such a design framework opens up the possibility of constructing rapid, controllable reaction circuits using four-way branch migration in the field of dynamic nucleic acid nanotechnology.

## Introduction of a Single Bulge

We hypothesized that the presence of a bulge (unpaired base) at the Holliday junction should be sufficient to trigger a faster four-way branch migration mechanism, with increased reaction kinetics (Figure 2a). A bulge at the junction can minimize the thermodynamic penalty associated with each branch migration step, as the opening of a single base pair is sufficient for branch migration initiation. Also, the bulge should destabilize the stacked form of the Holliday junction to further facilitate branch migration. We visualize the progression of a four-way branch migration in the presence of a bulge in Figure 2a. In state 1, a bulge forms adjacent to the toeholds between arms 1 and 3 of the DNA junction, after toehold hybridization. Thermal fluctuations allow a base pair in arm 4 to open (state 2). The revealed base *n*′ can then bind to the free nucleotide at the bulge (*n*), forming a new base pair. Overall, this process causes arm 4 to be reduced in length by one base pair, while arm 1 is extended correspondingly. We refer to this step from state 1 to state 3 as a half-migration step, as only two of the arms have been altered. A second half-migration step, led by the opening of a base pair in arm 3 (state 4), completes a single branch migration step. At the end of this step (state 5), the junction and bulge have migrated by one step, and the bulge returns to its original position relative to the four-way junction.

**Figure 2:**
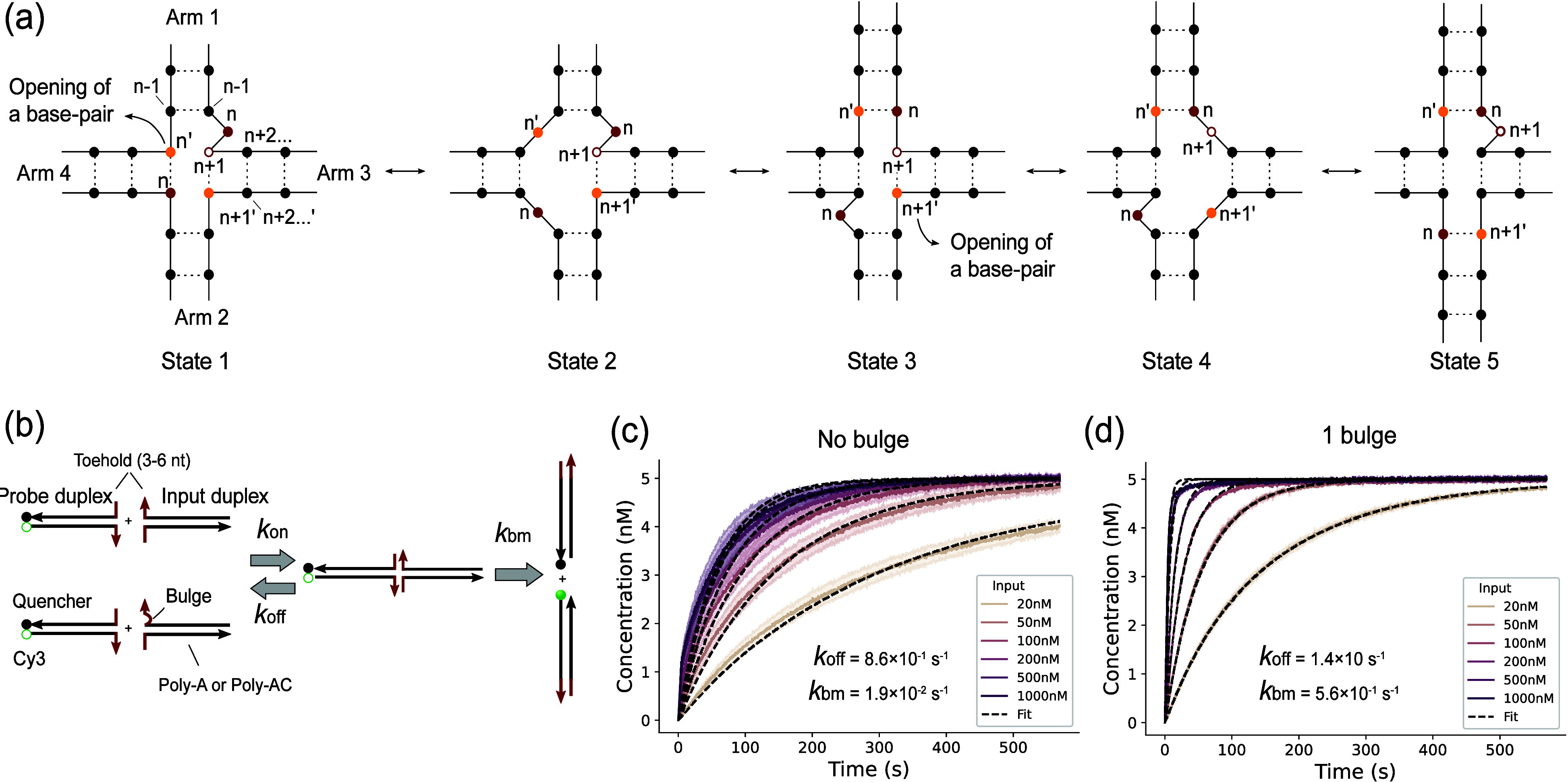
Four-way strand exchange with a single bulge. (a) Proposed molecular states during four-way strand exchange with a single bulge. (b) Experimental design to compare the four-way branch migration reaction kinetics in the presence and absence of a single bulge. Complete strand displacement recovers a fluorescent product that acts as a reporter of the reaction kinetics. Rate constants for our three-parameter model of four-way branch migration (*k*_on_, *k*_off_, and *k*_bm_) are also indicated. (c and d) Normalized fluorescent traces obtained by combining 5 nM probe duplex and 20–1000 nM input duplex in the (c) absence or (d) presence of a single bulge. Shaded regions represent the standard deviation calculated from two independent experiments. Black, dashed lines represent the fitted curves using the three reaction parameters in panel b with only a single set of parameters used for the six different concentrations.

In order to assess the effect of introducing a bulge on the kinetics of a four-way branch migration, we employed a simple design involving two DNA duplexes (Figure 2b and Table S1). Each strand in the first duplex, known as the input duplex, has a 5 nt toehold that is complementary to one toehold on the second duplex, known as the probe duplex. The probe duplex has a fluorophore (Cy3) and a quencher, which separate upon completion of the strand exchange reaction. An increase in the Cy3 fluorescence is used to estimate the kinetics of this four-way branch migration process. In addition, we designed a blocker strand to limit undesired three-way branch migration reactions coming from any single-stranded species that could be present in the solution (see the Materials and Methods for details and Figure S1 for kinetic traces without blockers).

When we mixed different concentrations of input duplex ranging from 20 to 1000 nM, with 5 nM probe duplex, the input duplex with a bulge exhibited much faster strand exchange (Figure 2c and d), particularly at high concentrations. Notably, as we increased the concentration of input strand up to 1000 nM, we observed the reaction kinetics becoming saturated. This is in line with our expectation, wherein the binding reaction is fast at a high concentration and the branch migration rate becomes the rate-limiting step. While the reaction saturated for both the standard reactions and those with bulges, the presence of bulges pushed the speed limit of the strand exchange reactions to higher rates. The rate-limiting step at a higher concentration, branch migration, has thus been accelerated. A similar behavior was observed for a different set of toeholds (Figure S2).

## Introduction of Two Bulges

We next tested the effect of adding a second bulge at the Holliday junction to see if our mechanism can be generalized and if the speed limit can be further increased by further destabilizing the junction. As in the single-bulge case, we hypothesize that the mechanism of four-way branch migration with two bulges involves five states (Figure 3a). In state 1, the hybridization of the toeholds results in the formation of a bulge (at base *n*) between arms 1 and 3 and a second bulge (at base *n* – 1) between arms 2 and 4. Notably, opening of a single base pair in either arm 2 or 3 should be able to initiate a half-migration step. Assuming a base pair opens in arm 4 (state 2), a new base pair can form in arm 1 and a 2 nt bulge forms between arms 2 and 4 (state 3). We refer to this step as a half-migration step. In state 4, a base pair opens in arm 3, facilitating the formation of a new base pair with one nucleotide in the 2 nt bulge (state 5). One complete strand displacement step regenerates two bulges at the original positions relative to the four-way junction.

**Figure 3:**
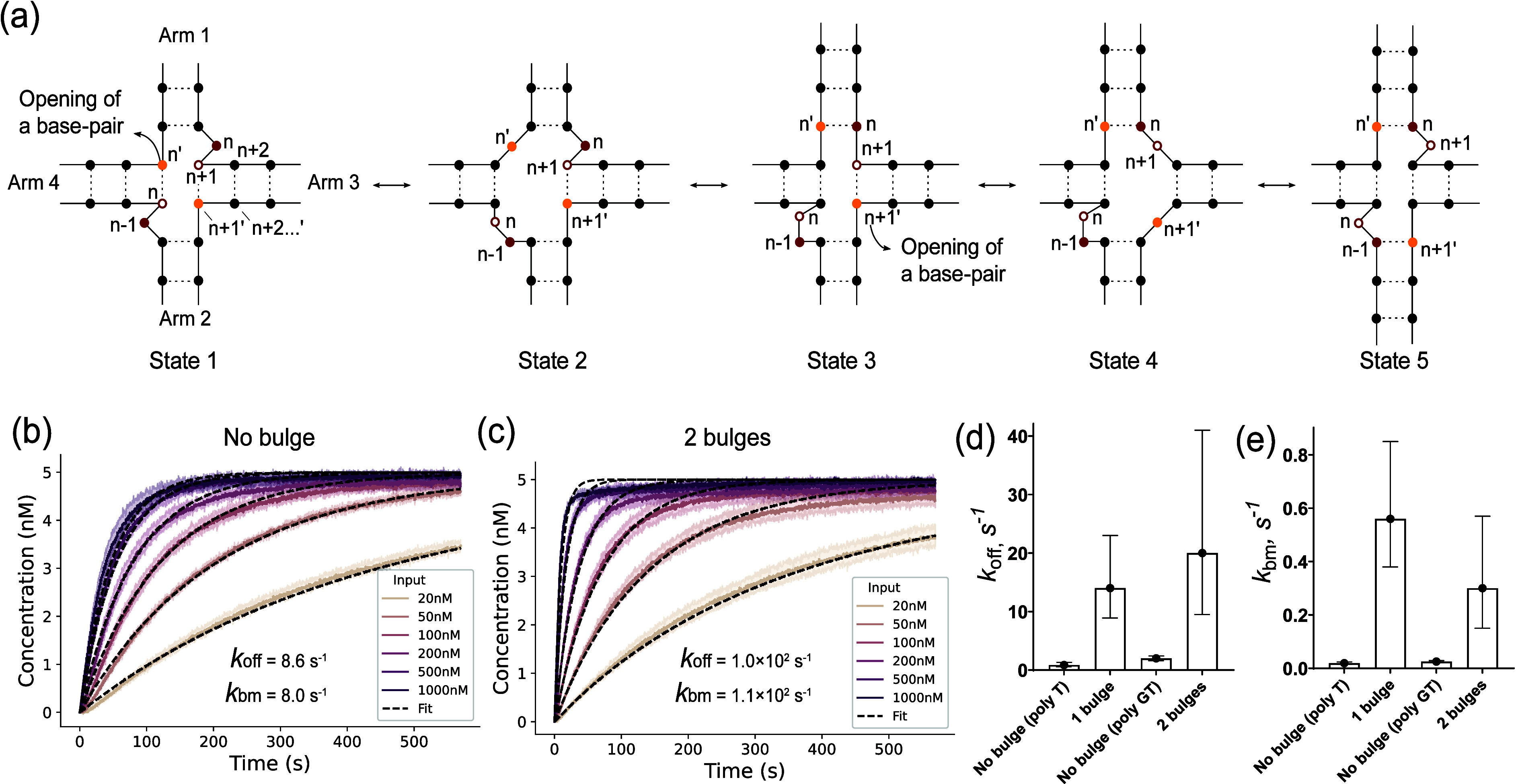
Effect of two bulges on four-way branch migration kinetics and characterization of kinetics. (a) Proposed molecular states during four-way strand exchange with two bulges. (b and c) Normalized fluorescent traces from combining 5 nM probe duplex and 20–1000 nM input duplex in the (b) absence or (c) presence of two bulges. (d) *k*_off_ and *k*_bm_ values from the fitting. Error bars represent uncertainties from the fitting.

Comparing the kinetics of input duplexes with and without two bulges, we observed once again that the presence of two bulges accelerated the strand exchange reaction and pushed the speed limit of the reactions to higher rates (Figure 3b and c). A similar effect was observed when we tested a different set of toeholds (Figure S3).

## Fitting of the Strand Exchange with a Three-Parameter Model

To further characterize the kinetics of our four-way strand displacement systems, we considered a three-parameter model to describe the reaction kinetics. We define *k*_on_, *k*_off_, and *k*_bm_ as the rate constants of bimolecular binding, unimolecular unbinding, and branch migration, respectively (Figure 2b). Note that, in doing so, we approximate the branch migration process as a single step; this level of detail has proven sufficient to describe systems like ours.^19^ We modeled the reaction kinetics using a system of ordinary differential equations (ODEs) that describe the time-dependent concentrations of five species: input strand, probe, toehold-bound intermediate (*t*_only_), fluorescent product (*F*), and quenched complex (*Q*).

1 $$\frac{d\left[\mathrm{input}\right]}{dt}=k_{\mathrm{off}}\left[t_{\mathrm{only}}\right]-k_{\mathrm{on}}\left[\mathrm{input}\right]\left[\mathrm{probe}\right]$$

2 $$\frac{d\left[\mathrm{probe}\right]}{dt}=\frac{d\left[\mathrm{input}\right]}{dt}$$

3 $$\frac{d\left[t_{\mathrm{only}}\right]}{dt}=k_{\mathrm{on}}\left[\mathrm{input}\right]\left[\mathrm{probe}\right]-k_{\mathrm{off}}\left[t_{\mathrm{only}}\right]-k_{\mathrm{bm}}\left[t_{\mathrm{only}}\right]$$

4 $$\frac{d\left[F\right]}{dt}=k_{\mathrm{bm}}\left[t_{\mathrm{only}}\right]$$

5 $$\frac{d\left[Q\right]}{dt}=\frac{d\left[F\right]}{dt}$$

The equations capture reversible toehold binding and the irreversible formation of the fluorescent product via branch migration, enabling quantitative fits to experimental data.

As the difference in concentrations of input complex should not affect the rate constants, we fit all curves in the same panel with the same set of parameters (four sets of parameters for Figures 2c and d and 3b and c). Although the quality of the fitting was good, we found that not all of the individual fittings provided reliable rate estimates. This was because not all parameters were independently constrained by the available data (Supplementary Note 1). We therefore tried to reduce the parameter space by fixing one of the parameters. We found that fitting *k*_off_ and *k*_bm_ while fixing *k*_on_ to the previously reported maximum rate of hybridization, 10^7^ M^–1^ s^–1^,^32^ gave reasonable values and good fits to all six kinetic curves in each graph (dashed lines in Figures 2c and d and 3b and c). We confirmed that our conclusions are not sensitive to our assumption that *k*_on_ = 10^7^ M^–1^ s^–1^ by systematically altering the fixed *k*_on_ value and evaluating its effect on the *k*_bm_ estimate. Across all experiments, we estimated a similar branch migration rate constant (within 2.5%) for a range of fixed *k*_on_ values from *k*_on_ = 10^6^ M^–1^ s^–1^ to 10^8^ M^–1^ s^–1^ (Supplementary Note 1 and Figure S4). For single-bulge experiments, *k*_off_ was estimated at 1.4 × 10^–1^ s^–1^, with a 95% confidence interval (CI) [89, 23.0], and 8.6 × 10^–1^ s^–1^, with a 95% CI [0.55, 1.3], in the presence and absence of a bulge, respectively (Figure 3d). The mean *k*_bm_ estimate was 5.6 × 10^–1^ s^–1^, with a 95% CI [0.38, 0.85], and 1.9 × 10^–2^ s^–1^, with a 95% CI [0.015, 0.024], in the presence and absence of a bulge, respectively (Figure 3e). Thus, we concluded that the presence of a bulge accelerated the branch migration process and increased the level of unbinding of the toeholds.

We estimated the values of *k*_off_ and *k*_bm_ for two-bulge experiments as well. The mean *k*_off_ estimate was 1.0 × 10^2^ s^–1^, with a 95% CI [1.5, 6.7 × 10^3^], and 8.0 × 10^0^ s^–1^, with a 95% CI [0.47, 1.4 × 10^1^], in the presence and absence of the two bulges, respectively (Figure 3d). We estimated mean *k*_bm_ values of 1.1 × 10^2^ s^–1^, with a 95% CI [1.7, 7.6 × 10^3^], and 1.6 × 10^0^ s^–1^, with a 95% CI [0.16, 17], in the presence and absence of the two bulges, respectively (Figure 3d). Similar to the single-bulge system, the presence of two bulges destabilized the four-way DNA junction while significantly increasing the branch migration rate. We observed a minor deviation between the data and the fitting in Figure 3b and c, likely due to errors in the probe concentration from pipetting or the presence of truncated DNA from synthesis, which could lead to a slower, tailed response.

For both the single- and double-bulge cases, the increase in *k*_bm_ and increase in *k*_off_ have largely compensatory effects when the concentration of the probe is low and binding events are rare. Probes with bulges can perform branch migration faster but spend less time bound, and therefore, the probability of successful displacement during a single-probe binding event is similar to probes without bulges. Since both bind at a similar rate, the overall reaction rate is similar, as can be seen in Figures 2 and 3. When the probe concentration is high, however, binding events are rapid, and therefore, the actual amount of time spent in the four-stranded complex becomes significant in determining reaction kinetics, favoring the designs with bulges in which the resolution of the Holliday junction is much faster. At the highest concentrations, the process becomes effectively first-order with a rate entirely determined by *k*_bm_. The systems without bulges clearly show this effect in Figures 2c and 3b; the increasing input concentration has an ever-decreasing return in terms of the reaction rate, tending toward a limiting curve with a relatively low rate set by *k*_bm_. By contrast, the systems with bulges in Figures 2d and 3c exhibit much higher limiting reaction rates.

## Thorough Characterization of Four-Way Strand Exchange Kinetics in the Presence of Bulge(s)

To further quantify how the bulges accelerate the four-way strand exchange reactions, we have tested toeholds with lengths from 3 to 6 nt with input duplex concentrations ranging from 20 to 1000 nM in the presence and absence of one (design 2) or two (design 1) bulges. Similar to the previous experiments with 5 nt toeholds, we have observed acceleration of the four-way strand exchange reactions in every concentration that we tested (Figures S2, S5, and S6).

However, attempting to fit all of the reactions did not provide reliable rate estimates. To provide a model-free quantification of the effect of bulges on strand displacement, we report the time for 90% reaction completion for each toehold length and input duplex concentration (Figure 4a and b, Figure S6, and Materials and Methods). This procedure was effective for toeholds longer than 3 for one-bulge systems and toeholds longer than 4 for two-bulge systems. In these cases, we observe up to 16-fold acceleration for the longest toeholds at the highest concentrations and generally observed higher acceleration as the toehold length and concentration of the input duplex increased (Figure 4c). This result is consistent with our proposed mechanism that bulge accelerates the branch migration reactions, which becomes the rate-limiting step for long toeholds and high concentrations, although we do note that two-bulge systems do show a surprisingly high acceleration for shorter toeholds. We anticipate toeholds with a greater GC content will behave like longer toeholds due to their more negative free energy of binding, as is observed for three-way strand displacement.^18,19^

**Figure 4:**
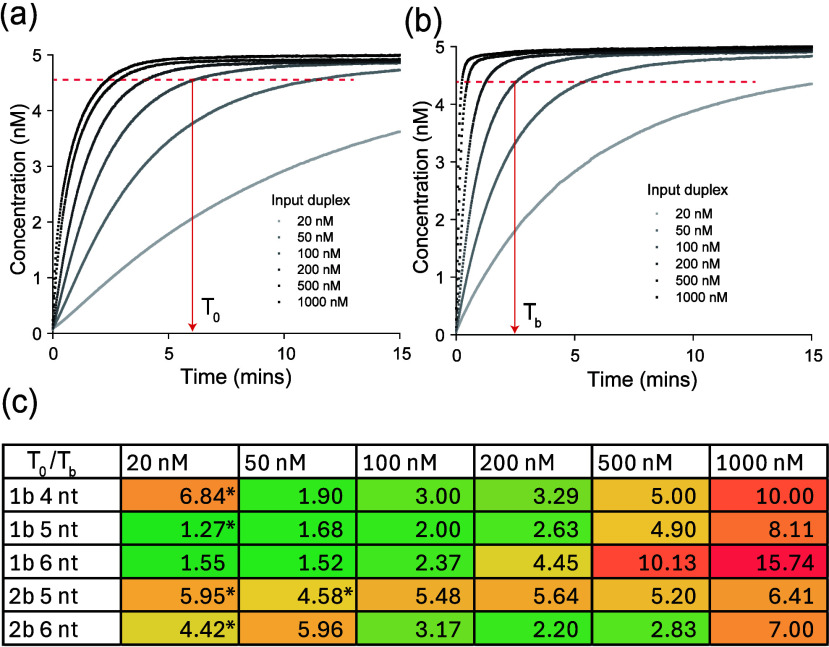
Reaction enhancement factor. (a and b) Exemplar plots indicating 90% reaction data points for the 5 nt toehold reaction in the (a) absence, *T*_0_, and (b) presence, *T*_b_, of a bulge. (c) Reaction enhancement factor (*T*_0_/*T*_b_) for different lengths of toeholds and concentrations of input duplexes. Please see Supplementary Note 2 for a description of the rates with ∗.

## oxDNA Simulation

Although the introduction of bulges successfully increased the speed limit of the strand exchange reaction, the increase in speed was not as big as we expected from a picture in which the bulges both reduce the number of base pairs that must be disrupted at any one time and destabilize the relatively immobile, stacked junction conformation. This unexpected behavior was particularly notable for the double-bulge system, which had a smaller increase in speed than for the single-bulge system overall. We therefore performed oxDNA simulations to further understand the molecular behavior of our system (Figure 5a).^31,33^ The simulations were set up and run for comparable experimental conditions, temperature, salt concentration, and sequences, but did not include the Cy3 fluorophore–quencher pair, which is common in both conditions.

**Figure 5:**
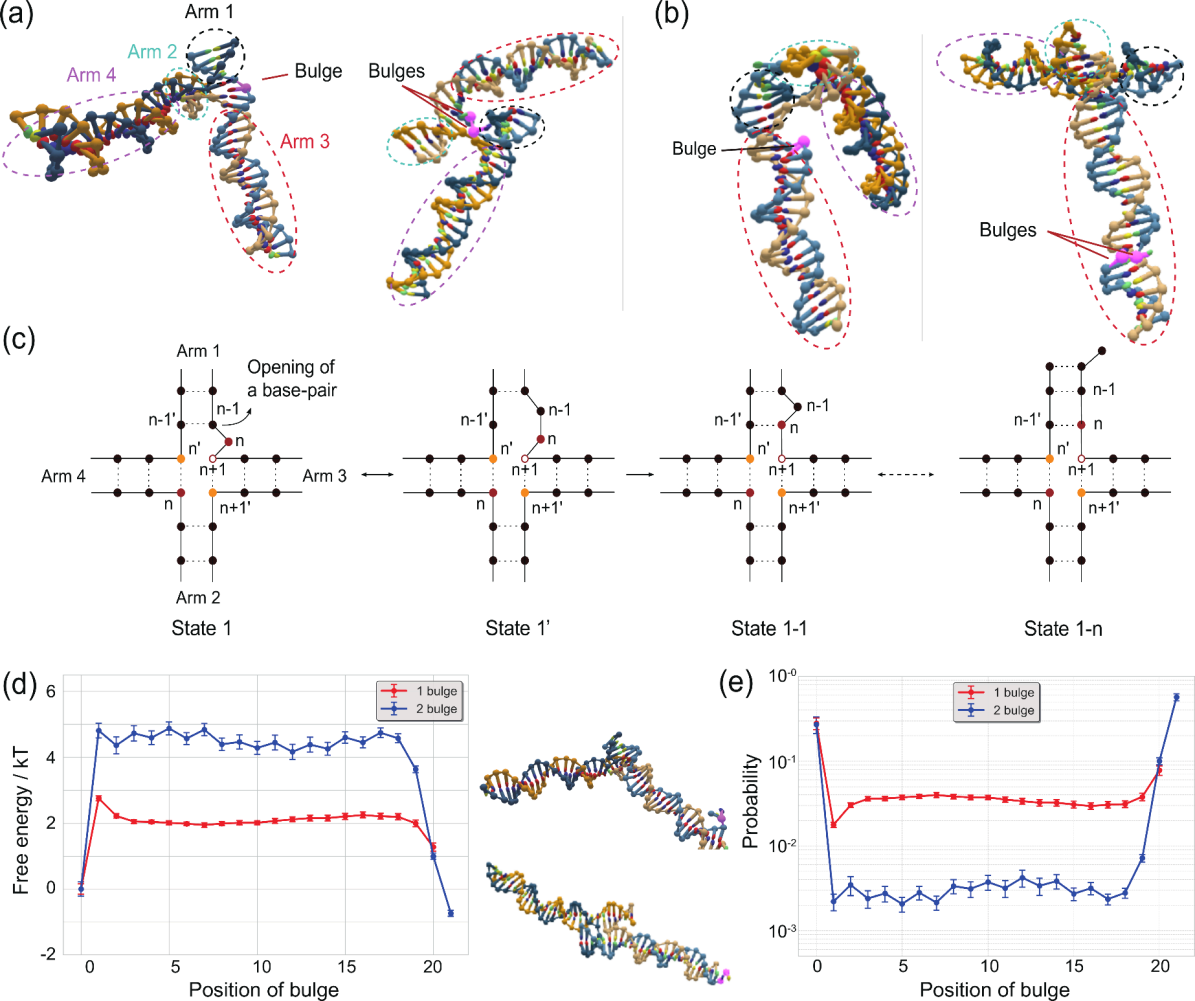
oxDNA predictions of bulge migration in the absence of branch migration. For the one- and two-bulge systems, oxDNA simulations were initiated with probe and input bound by toeholds alone. (a and b) Representative images of the Holliday junction where the bulges are (a) at the toehold, as initiated, and (b) in the middle of branch migration domains, as observed later in simulations. (c) Proposed states for the diffusion of bulges with a fixed junction. (d) Probability distribution of the position of the bulge(s) in the displacement domain of the one- and two-bulge systems, given a fixed junction location. Location 0 on the horizontal axis represents the bulge present near the junction, while the rightmost point (20 for the one-bulge system and 21 for the two-bulge system) represents the bulge beyond the final position in the displacement domain (where the duplex can enter the frayed state). Representative images of the Holliday junction when the bulges are at location 0 are shown on the right. (e) Free energy $\frac{F\left(x\right)}{kT}=-\ln\frac{P\left(x\right)}{P\left(1\right)}$ as a function of bulge position *x* where *p*(*x*) is the probability of finding the bulge at position *x*.

During initial oxDNA simulations, it was observed that the bulge not only participates in the branch migration process but can also diffuse internally along the arm of the input or probe duplex (Figure 5b). While this bulge diffusion does not block the branch migration process, it eliminates the accelerating effect of the bulge when the bulge is not in the correct position at the junction. For the system with a single bulge, this diffusion is illustrated schematically in Figure 5c. When in state 1, the base pair (*n* + 1, *n* + 1′) of arm 3 or (*n* – 1, *n* – 1′) of arm 1 can open before the base pair (*n*, *n*′) of arm 4 (Figure 5c, state 1′), and the bulge can diffuse away from the junction. Due to the free-energy penalty of incorporating a bulge within the interior of a duplex, this diffusion is unlikely to happen when the input and probe duplexes are isolated. However, when the duplexes bind to form a junction, those extra bases now destabilize the junction by design. Thus, the relative cost of allowing the bulge to diffuse into the interior of the duplex is much lower. A step of diffusion for the bulge is complete when the new base pair (*n*, *n* + 1′) regenerates the (*n* + 1) base of arm 3 as the new bulge (Figure 5c, state 1-1). Similar to branch migration, this reversible process can iterate until the end of the branch migration domain (Figure 5c, state 1-*n*).

To explicitly study this bulge diffusion, we ran oxDNA simulations, while allowing bulge diffusion but prohibiting branch migration. Doing so, we observed the bulge diffusing through the duplex (Figure 5d and e) with two preferred locations: one near the junction and the other at the far end of the duplex arm (Figure 5d, right images). The one-bulge system exhibits a slightly higher probability of staying near the junction (0.28 for one bulge and 0.156 for two bulges), which explains why the increase in speed was not as high as expected. In the two-bulge system, the bulges have a high probability of being found at the far end, which may contribute significantly to reducing the branch migration rate. Moreover, the existence of a long-lived low mobility state may explain why the high concentration curves in Figure 3c show anomalously slow convergence to the maximum signal in the final stages of the reaction.

Herein, we present an alternative design for four-way DNA branch migration systems with a higher speed limit, up to 16-fold faster than conventional systems. This increase in speed comes from the introduction of a bulge at the Holliday junction, which increases the branch migration rate. Consequently, this work offers a reaction scheme with improved kinetics, applicable to a number of nucleic acid reaction networks that rely on four-way branch migration, including molecular walkers^34^ and DNA actuators.^35^ We expect that our scheme would be particularly useful with high local concentrations of species, such as in reactions on DNA origami or within molecular condensates.

We note that the introduction of a single bulge enforces a constraint on the sequence of the entire branch migration domain as base *n* must be complementary to base *n* + 1′, as is evident from state 5 of Figure 3a (Figure S7). Consequently, the branch migration domain is limited to poly-A or poly-T sequences, as poly-G sequences cannot be synthesized. The presence of a second bulge reduces the sequence constraints on the displacement domain as now *n* – 1 must only be complementary to *n* + 1′ throughout the displacement domain, resulting in poly-GT/poly-CA sequences. Nevertheless, the toehold domains are not subjected to a similar constraint, and thus, our design remains compatible with the majority of four-way branch migration reaction networks proposed thus far. Indeed, Johnson and Qian have pointed out that the ability to use orthogonal branch migration domains in four-way strand displacement networks is inherently limited anyway.^22^ However, for applications involving external or naturally occurring sequences, these sequence constraints pose challenges. In principle, larger circuits involving multiple coupled reactions can be constructed using a four-way toehold exchange.^36^ Here, the bulge motif would be implemented in cascades of reactions that use the same branch migration domains but which result in novel pairs of toeholds, allowing products to undergo novel reactions.^37^

In this work, we have used a simple three-state, kinetic model to rationalize and quantify the observed behavior. While this model does a reasonable job of predicting qualitative trends, with bulges either neutral or accelerating the reaction with strong acceleration in the saturated limit, there are anomalies that are hard to explain, such as the relative speed of 4 nt, two-bulge systems and the apparent slowness of some no-bulge systems. In future work, it would be beneficial to develop multi-state models of the branch migration, along the lines of those used in three-way branch migration,^19,38^ to see if a model that quantitatively predicts acceleration factors is possible.

oxDNA simulations explained why *k*_bm_ did not increase as much as might have been expected. oxDNA simulations show that the bulge(s) can diffuse along the displacement domain, a process which competes with accelerated junction migration. When the bulge is not at the junction core, the junction migrates as if there is no bulge. Whenever the diffusing junction meets the diffusing bulge during the displacement process, accelerated migration ensues.

As mentioned in the introduction, the absence of single-stranded species makes four-way branch migration ideal for *in vivo* application due to a reduction in cross-talk and higher stability.^28^ The recently developed protocol for autonomously generating multi-stranded nucleic acid species^39,40^ could, in principle, be extended to four-way branch migration reaction schemes. We expect to combine both methods to implement an optimized four-way branch migration scheme *in vivo* as part of future work.

## Supplementary Material



## Data Availability

Raw data, fitting code, and oxDNA simulation files are freely available at 10.5281/zenodo.15398317.

## Notes

Materials and Methods, Supplementary Notes 1 and 2, Table S1, and Figures S1–S7 (PDF)
