# Supplementary material for: Overcoming the Speed Limit of Four-Way DNA Branch Migration with Bulges in Toeholds
Source: Nano Lett. 2025 Sep 4;25(37):13772–9. doi: 10.1021/acs.nanolett.5c03063 (PMC12447554; doi:10.1021/acs.nanolett.5c03063)
Supplement: Supplementary file 1 [file nl5c03063_si_001.pdf]

Supplementary material for

Overcoming the speed limit of four-way DNA branch migration with bulges in toe-holds

Samia Bakhtawar‡[1], Francesca Smith[2], Aditya Sengar[2], Guy-Bart V. Stan[2], John Goertz[3], Molly Stevens[3,4], Thomas E. Ouldridge[*2] and Wooli Bae‡[*1]

[1]School of Mathematics and Physics, Faculty of Engineering and Physical Sciences, University of Surrey, Guildford GU2 7XH, U.K.

[2]Imperial College Centre for Synthetic Biology and Department of Bioengineering, Imperial College London, South Kensington Campus, London SW7 2AZ, U.K.

[3]Department of Materials, Department of Bioengineering and Institute of Biomedical Engineering, Imperial College London, London, SW7 2AZ, U.K.

[4]Department of Physiology, Anatomy and Genetics, Department of Engineering Science, Kavli Institute for Nanoscience Discovery, University of Oxford, Oxford, OX1 3QU

‡S.B. and W.B. contributed equally to the manuscript

*Email: t.ouldridge@imperial.ac.uk

*Email: w.bae@surrey.ac.uk

Table of contents

Materials and methods

Supplementary note S1

Table S1

Figure S1

Figure S2

Figure S3

Figure S4

Figure S5

Figure S6

Materials and methods

DNA sequence design

All sequences were designed using NUPACK and ordered from Integrated DNA Technologies (IDT). All strands were ordered with HPLC purification at 100 µM in LabReady IDTE buffer (pH 8.0). The sequences used in this work are given in Table 1.

Table S1. Experimental sequence design. Bulges are shown in blue; the toehold is shown in red and bulges are marked in blue. Cy3 and FQ Iowa Black were used as fluorophore and quencher, respectively. Sequences are given 5' to 3'.

Name	Sequence	Used in Figure(s)
Input A (5nt)	5`-CTA AAA AAA AAA AAA AAA AAA AGG GAA-3`	Figure 2c, 2d, S1
Input T (5nt)	5`-AGG AGT TTT TTT TTT TTT TTT TTT TAG-3`	Figure 2c, S1
Blocker T	5`-TTT TTT TTT TTT TTT TTT TTA G-3`	Figure 2c, 2d, 4a, 4b, 4c, S1, S2
Probe F-A (6nt)	5`-/fCy3/GTA AAA AAA AAA AAA AAA AAA ACT CCT T-3`	
Probe F-A (5nt)	5`-/fCy3/GTA AAA AAA AAA AAA AAA AAA ACT CCT-3`	Figure 2c, S1
Probe Q-T (6nt)	5`-CTT CCC TTT TTT TTT TTT TTT TTT TTA C/3IABkFQ/-3`	
Probe Q-T (5nt)	5`-TT CCC TTT TTT TTT TTT TTT TTT TTA C/3IABkFQ/-3`	Figure S1
Input T 1b (5nt)	5`-AGG AGT TTT TTT TTT TTT TTT TTT TTA G-3`	Figure 2d, S1
Input CA (6nt)	5`-GAC ACA CAC ACA CAC ACA CAC GCG AAA-3`	Figure 4c, S5
Input TG (6nt)	5`-GAG AAA GTG TGT GTG TGT GTG TGT GTC-3`	Figure 4c, S5
Input CA (5nt)	5`-GAC ACA CAC ACA CAC ACA CAC GCG AA-3`	Figure 4c, 3b, 3c, S4
Input TG (5nt)	5`-AGA AAG TGT GTG TGT GTG TGT GTG TC-3`	Figure 4c, 3b, S4
Input CA (4nt)	5`-GAC ACA CAC ACA CAC ACA CAC GCG A-3`	Figure S5
Input TG (4nt)	5`-GAA AGT GTG TGT GTG TGT GTG TGT C-3`	Figure S5
Input CA (3nt)	5`-GAC ACA CAC ACA CAC ACA CAC GCG-3`	Figure S5
Input TG (3nt)	5`-AAA GTG TGT GTG TGT GTG TGT GTC-3`	Figure S5
Probe Q-TG (6nt)	5`-TTT CGC GTG TGT GTG TGT GTG TGT GTG/3IABkFQ/-3`	Figure 4c, S5
Blocker TG	5`-GTG TGT GTG TGT GTG TGT GTC-3`	Figure 3b, 3c, 4c, S2, S3, S4, S5
Probe F-CA (6nt)	5`-/5Cy3/CAC ACA CAC ACA CAC ACA CAC TTT CT-3`	Figure 3b, 4c, S5

Input CA 2b (6nt)	5`-GAC ACA CAC ACA CAC ACA CAC AGC GAA A-3`	Figure 4c, S5
Input CA 2b (5nt)	5`-GAC ACA CAC ACA CAC ACA CAC AGC GAA-3`	Figure 3c, 4c, S3
Input CA 2b (4nt)	5`-GAC ACA CAC ACA CAC ACA CAC AGC GA-3`	Figure S5
Input CA 2b (3nt)	5`-GAC ACA CAC ACA CAC ACA CAC AGC G-3`	Figure S5
Probe F-CA 2b (6nt)	5`-/fCy3/CCA CAC ACA CAC ACA CAC ACA ACT TTC T-3` (to check)	Figure 3c, 4c, S5
Probe Q-TG 2b (6nt)	5`-TTT CGC TGT GTG TGT GTG TGT GTG TGG/3IABkFQ/-3`	Figure 3c, 4c, S5
	Design 2	
Input A2 (6nt)	5`-CTA AAA AAA AAA AAA AAA AAA AGT TCC G-3`	Figure 4c, S2
Input A2 (5nt)	5`-CTA AAA AAA AAA AAA AAA AAA AGT TCC-3`	Figure 4a, 4b, 4c, S2
Input A2 (4nt)	5`-CTA AAA AAA AAA AAA AAA AAA AGT TC-3`	Figure 4c, S2
Input A2 (3nt)	5`-CTA AAA AAA AAA AAA AAA AAA AGT T-3`	Figure S2
Input T2 (6nt)	5`-GCA TCG TTT TTT TTT TTT TTT TTT TTA G-3`	Figure 4c, S2
Input T2 (5nt)	5`-CAT CGT TTT TTT TTT TTT TTT TTT TAG-3`	Figure 4a, 4c, S2
Input T2 (4nt)	5`-AT CGT TTT TTT TTT TTT TTT TTT TAG-3`	Figure 4c, S2
Input T2 (3nt)	5`-T CGT TTT TTT TTT TTT TTT TTT TAG-3`	Figure S2
Input T2 1b (6nt)	5`-GCA TCG TTT TTT TTT TTT TTT TTT TTT AG-3`	Figure 4b, 4c, S2
Probe F-A2 (6nt)	5`-/5Cy3/GTA AAA AAA AAA AAA AAA AAA ACG ATG C-3`	Figure 4a, 4b, 4c, S2
Probe Q-T2 (6nt)	5`-CGG AAC TTT TTT TTT TTT TTT TTT TTA C/3IABkFQ/-3`	Figure 4a, 4b, 4c, S2
Input CA2 (5nt)	5`-GAC ACA CAC ACA CAC ACA CAC GTT CC-3`	Figure S3
Input TG2 (5nt)	5`-CAT CGG TGT GTG TGT GTG TGT GTG TC-3`	Figure S3
Probe F CA2 (5nt)	5`-/5Cy3/CAC ACA CAC ACA CAC ACA CAC C-3`	Figure S3
Probe Q TG2 (5nt)	5`-GGA GCG TGT GTG TGT GTG TGT GTG TG/3IABkFQ/-3`	Figure S3
Input CA2-2b (5nt)	5`-GAC ACA CAC ACA CAC ACA CAC AGT TC-3`	Figure S3
Probe F CA2-2b (5nt)	5`-/5Cy3/CCA CAC ACA CAC ACA CAC ACA C-3`	Figure S3

Probe Q TG2-2b (5nt)	5`-GGA ACT GTG TGT GTG TGT GTG TGT GG/3IABkFQ/-3`	Figure S3

DNA duplex annealing

Input and probe duplexes were prepared by annealing complementary strands, with the A-strands added in excess. Specifically, poly T (or TG) strands were mixed with poly A (or AC) strands at final concentrations of 4 µM and 4.1 µM respectively in 1X TE buffer containing 1 M NaCl. The annealing process was conducted using an Applied Biosystems ProFlex PCR thermal cycler. The samples were heated to 95°C for 5 minutes, followed by a gradual cooling to 25°C at a rate of 1°C every 2 minutes. After cooling, the samples were incubated at 25°C for 15 minutes, and then stored at 4°C. To finalise the preparation, 0.2 µM of poly T (or TG) blocker strands were added to the mixture and incubated at 45°C for 5 minutes to ensure proper integration (Figure S5).

Fluorescence spectroscopy

All fluorescence measurements were conducted using a Tecan microplate reader with Greiner 96 Flat Transparent Plates ([GRE96ft_CellCulture]). Reactions were carried out at a constant temperature of 25°C. To evaluate different concentrations of probe and input duplexes, probe duplexes were initially diluted to 6.7 nM. A total of 150 µl of the diluted probe duplex solution was injected using the Tecan Injector Module onto 50 µl of input duplexes at varying concentrations. The final reaction concentrations were 5 nM for probe duplexes and 20, 50, 100, 200, 500, and 1000 nM for input duplexes. After a brief 2-second mixing period by shaking, Cy3 emissions were recorded using excitation and emission wavelengths of 530/20 nm and 580/30 nm, respectively. The data was collected at 1s intervals for 6nt & 5nt and at 30s intervals for 4nt & 3nt, with the first measurement taken at 3.5 seconds. Background fluorescence from 5 nM of fully quenched probe duplexes was subtracted from the raw fluorescence values. Raw fluorescence values were converted to concentrations by dividing by fluorescence of un-quenched Cy3 duplex (ProbeF-A / input-T or ProbeF-CA / Input-TG duplexes). The fluorescence corresponding to 100% strand exchange was defined as the value obtained from 5 nM of the strand-exchanged product. Data for Figures 2c, d and 3b, c are the average of triplicates; all other data represent the average of two replicates. Typical averaged variability between the replica was less than 6% except for 3 nt design 2 with and without a bulge which was 30%, 22% in average.

Data fitting procedure

All fittings were performed in Python v3.8.8. We described the system using the following 5-ODE model, with rate constants k_{on}, k_{off} and k_{bm}:

$$\frac{d[Input]}{dt} = \frac{d[Probe]}{dt} = k_{\text{off}} \cdot [t_{\text{only}}] - k_{\text{on}} \cdot [Input] \cdot [Probe]$$

$$\frac{d[t_{\text{only}}]}{dt} = k_{\text{on}} \cdot [Input] \cdot [Probe] - k_{\text{off}} \cdot [t_{\text{only}}] - k_{\text{bm}} \cdot [t_{\text{only}}]$$

$$\frac{d[F]}{dt} = \frac{d[Q]}{dt} = k_{\text{bm}} \cdot [t_{\text{only}}]$$

We fit this ODE model to the experimental kinetic data to estimate values for the rate constants k_{off} and k_{bm}. k_{off} and k_{bm} were allowed to vary between $10^{-10}\ s^{-1}$ and $10^{10}\ s^{-1}$, with initial estimates of $10^{1}\ s^{-1}$. k_{off} and k_{bm} were estimated as log-transformed values to reduce the overall time to estima-

tion. k_{on} was restricted at $10^7\ M^{-1}\ s^{-1}$ to improve the overall fits. We employed a global fitting approach such that single values of k_{off} and k_{bm} were estimated for all six concentrations in each experiment (Figure 2c, d and Figure 3b, c). The final mean and standard error values reported for each were calculated by jackknife (leave-one-out) estimation. 95% confidence intervals are reported in the original units of measurement.

The Python code for fitting and estimating these rate constant estimates is available at: https://doi.org/10.5281/zenodo.7439302.

The fitting used following software packages –

- *SciPy v1.6.2 (scipy.optimize and scipy.integrate packages)*
- *jax v0.2.12 (jax.experimental.odeint package)*

OxDNA simulations

The oxDNA model is a coarse-grained model of DNA that can capture the structural, mechanical, and thermodynamic properties of DNA systems. For further details on how to set up and run the model, and other applications covered with oxDNA, we refer the interested reader to (37).

Simulation set-up

The simulations were performed in a box size of 70 units at 25 ºC at 1 M salt concentration. The 4 strands (2 input and 2 probe strands, Table 1) were loaded into the simulation box. Mutually attractive forces were applied (using the mutual traps feature in oxDNA) on the complementary bases of input-input and probe-probe strands to bring the system together. This was followed by applying mutually attractive forces on the complementary toeholds (inputA:probeQ-T input:probeF-A for the 1 bulge system and input-CA:probQ-TG and input-TG:ProbeF-CA for the 2-bulge system) to bind all the bases of the toehold. The fully bound configuration represents the starting system, which is similar to state 1 in Figure 2(a) for the one-bulge case, and state 3 in Figure 4(a) for the two-bulge case.

Simulations were performed with the built-in virtual move Monte Carlo algorithm in oxDNA. The system was allowed to equilibrate for 10^7 moves per particle before sampling was initiated. For each system, data was subsequently collected from 20 independent runs, corresponding to a total of 5.25 x 10^8 VMMC moves for the 1-bulge system and 5.43 x 10^8 VMMC moves for the 2-bulge system.

Bulge tracking and umbrella sampling

We will henceforth refer to the base pairs that are admissible when the bulge is near the junction as the *default* base pairs. When the location of the bulge is somewhere within the arm or at the far end, we will refer to the base pairs as *displaced* base pairs.

Simulations were run with the inbuilt umbrella sampling feature in oxDNA. Three types of parameters were defined to keep track of the number of bases formed. The first-order parameter (OP1) tracks all the default base pairs formed in the arm with the bulge (arm 1 of the one and two-bulge systems). The second-order parameter (OP2) tracks all the displaced base pairs formed. The third-order parameter (OP3) tracks all the possible base pairs of the other 3 arms. OP1 and OP2 have the same maximum value. Since we are studying the bulge diffusion process and not interested in observing junction migration, we prohibit the third-order parameter from having less than the maximum number of possible base pairs that it is tracking. Umbrella sampling is used to accelerate opening of base pairs, which helps increase the diffusivity of the bulge.

The location of the bulge at any time is tracked by the value of OP1 as long as OP1+OP2= n_{max} where n_{max} is the maximum possible number of default (or displaced) base pairs.

It was also observed that simulating the system with two bulges was relatively slow. To improve the sampling, we perform this simulation in 3 different windows. Each window has an overlap with the other window, allowing us to recover the entire landscape using the WHAM algorithm (38). The first window restricts the simulations with the condition: OP2≤5 such that the bulge diffuses close to the Holliday junction. The second window samples bulge diffusion for OP1≥4 and OP2≥4. Under this condition, the bulge never reaches either ends of the displacement domain. The third window samples bulge diffusion for OP2≤5. Under this condition, the bulge diffuses near the other end of the displacement domain.

All initialization files, including order parameter files and the weight files that correspond to the umbrella sampling simulations, are available at https://doi.org/10.5281/zenodo.15398317.

Supplementary note 1.

Fitting the three-parameter, two-step model to the experimental data was challenging, because the kinetics are not particularly sensitive to the absolute values of k_{on} and k_{off} individually. We therefore decided to fit the kinetics with a fixed $k_{\text{on}} = 10^7\ M^{-1}\ s^{-1}$. Relative values of k_{off}, then indicate toehold stability. Lower and upper bounds were set at $10^{-10}\ s^{-1}$ and $10^{10}\ s^{-1}$, respectively, for both k_{bm} and k_{off}. Then we assessed the effect of this choice of k_{on} on the key estimated value of k_{bm}. We determine the percentage change in the value of k_{bm} compared to the value estimated under the assumption of $k_{\text{on}} = 10^7\ M^{-1}\ s^{-1}$ for each experiment. We identify the value of k_{on} which incurs a percentage change of 2.5% in k_{bm}. For the no bulge system (20 nt poly-A/T branch migration domain), the lower bound is $k_{\text{on}} = 10^{5.47} M^{-1}\ s^{-1}$and although the exact upper bound was not identified it is greater than $k_{\text{on}} = 10^{8.80} M^{-1}\ s^{-1}$, which is already beyond the limit of reasonable k_{on} estimates. For the one-bulge system, the lower bound is $k_{\text{on}} = 10^{5.93} M^{-1}\ s^{-1}$ and although the exact upper bound was not identified it is greater than $k_{\text{on}} = 10^{8.50} M^{-1}\ s^{-1}$, which again is already beyond the limit of reasonable k_{on} estimates. For the no-bulge system (22nt poly-GT/CA branch migration domain), the lower bound is $k_{\text{on}} = 10^{5.28 M^{-1}\ s^{-1}}$ and although the exact upper bound was not identified it is greater than $k_{\text{on}} = 10^{8.80} M^{-1}\ s^{-1}$. For the two-bulge system, the lower bound is $\text{k}_{\text{on}} = 10^{6.20} M^{-1}\ s^{-1}$ and although the exact upper bound was not identified it is greater than $k_{\text{on}} = 10^{8.50} M^{-1}\ s^{-1}$. Across all experiments the value of k_{bm} differed by less than 2.5% between $k_{\text{on}} = 10^{6.2} M^{-1}\ s^{-1}$ and $k_{\text{on}} = 10^8 M^{-1}\ s^{-1}$. We assessed whether these alternative k_{on} values provided equivalently reasonable fits to the experimental data, and observed good fits to the curves at $k_{\text{on}} = 10^8\ M^{-1}\ s^{-1}$ as well as $10^7\ M^{-1}\ s^{-1}$.

Supplementary note 2 (for 4(c))

For some conditions the reaction did not reach 90% by the end of the measurement. In such cases, we measured the maximum level the slower reaction reached and then determined the time it took for the faster reaction to achieve that same level to measure the reaction enhancement factor. These data points are marked with an asterisk (*) in Figure 4c.

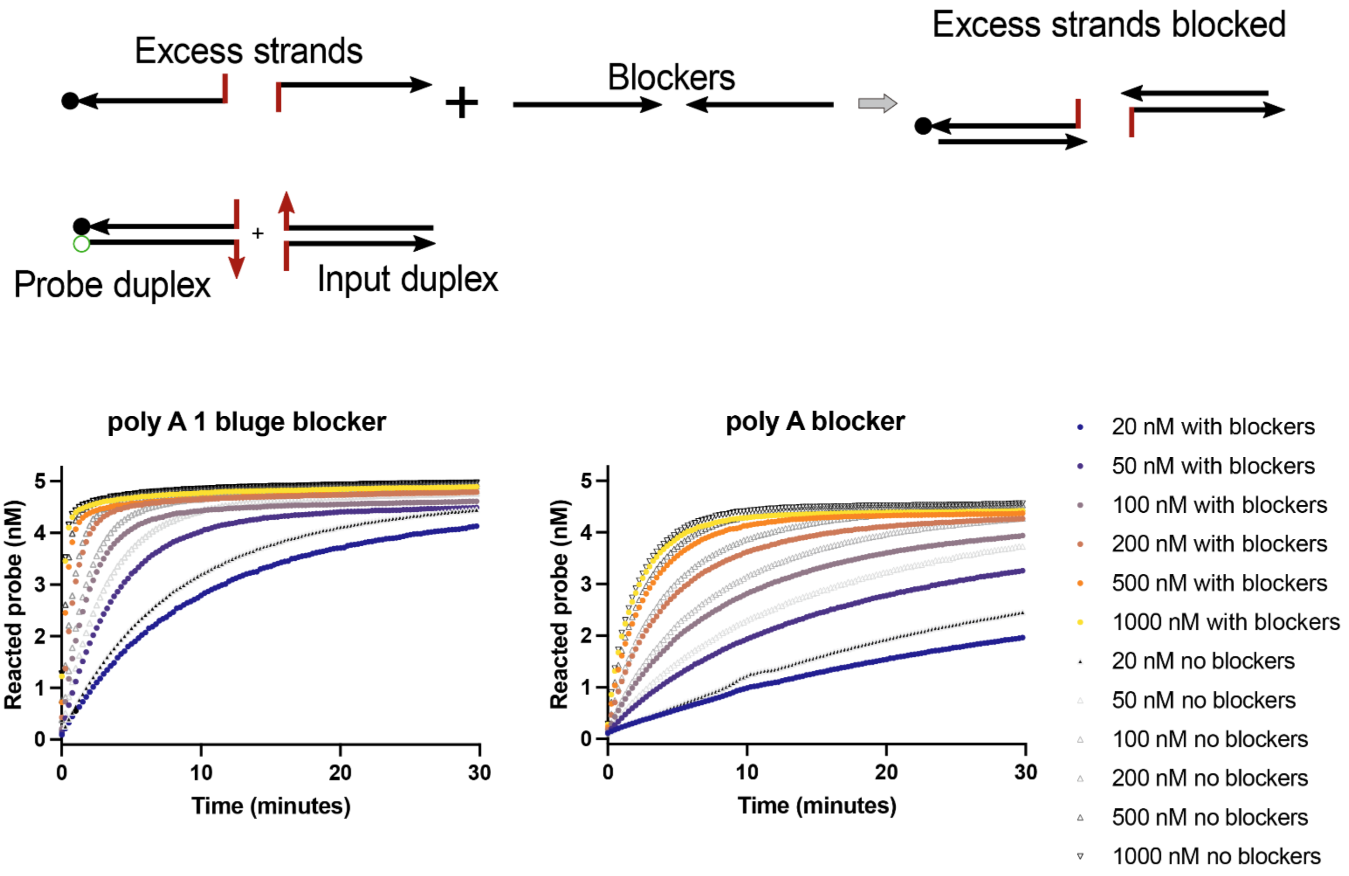

Figure S1. Graphical representation and the effect of blockers. Blockers are introduced as described in the Methods to prevent any 3-way branch migration reactions from excessive single-stranded DNA species. In the above, we compare the results of experiments performed with and without blockers, showing that although they make results cleaner, they are not necessary to observe acceleration in the presence of bulges (Greyscale data vs coloured data).

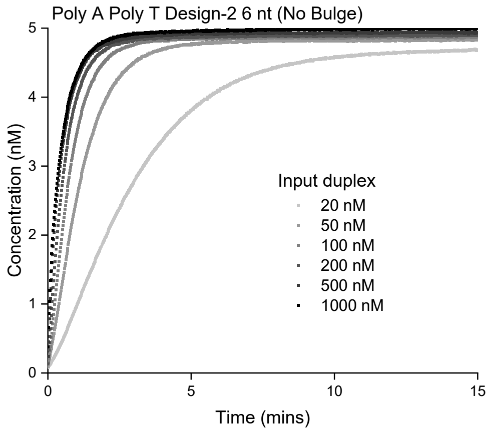
Poly A Poly T Design-2 6 nt (No Bulge)
Concentration (nM)
Time (mins)
Input duplex
20 nM
50 nM
100 nM
200 nM
500 nM
1000 nM

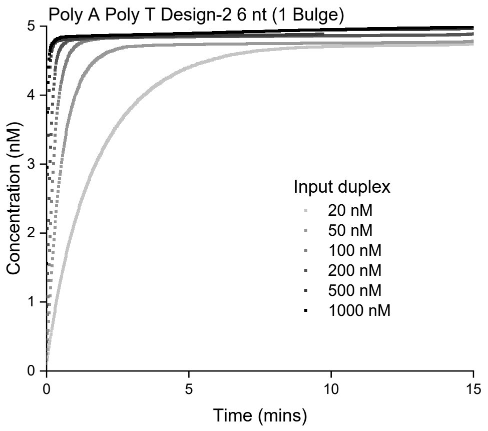
Poly A Poly T Design-2 6 nt (1 Bulge)
Concentration (nM)
Time (mins)
Input duplex
20 nM
50 nM
100 nM
200 nM
500 nM
1000 nM

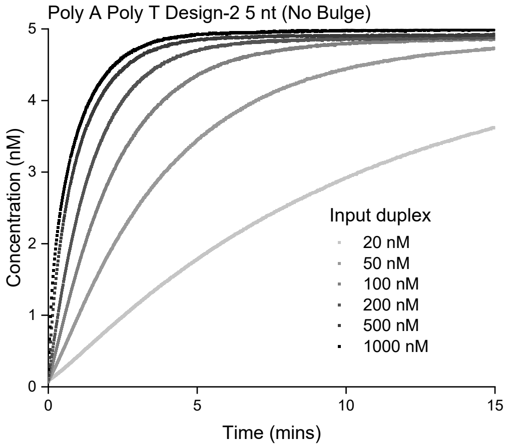
Poly A Poly T Design-2 5 nt (No Bulge)
Concentration (nM)
Time (mins)
Input duplex
20 nM
50 nM
100 nM
200 nM
500 nM
1000 nM

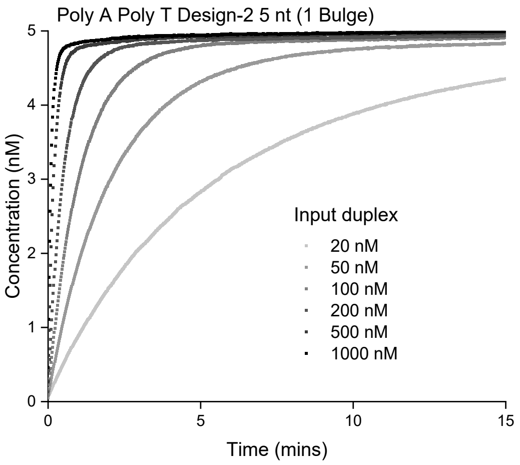
Poly A Poly T Design-2 5 nt (1 Bulge)
Concentration (nM)
Time (mins)
Input duplex
20 nM
50 nM
100 nM
200 nM
500 nM
1000 nM

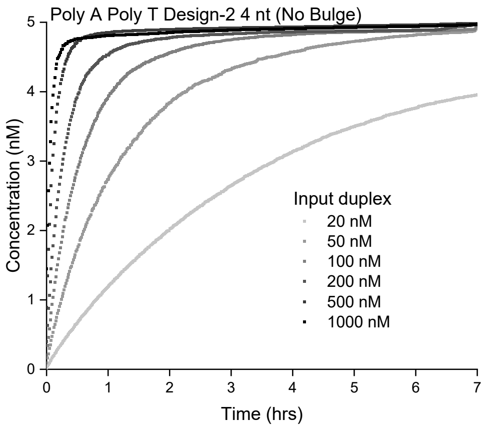
Poly A Poly T Design-2 4 nt (No Bulge)
Concentration (nM)
Time (hrs)
Input duplex
20 nM
50 nM
100 nM
200 nM
500 nM
1000 nM

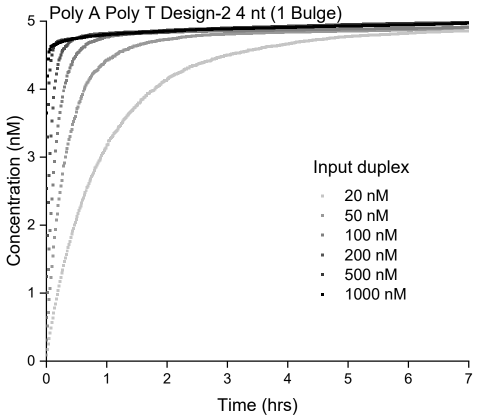
Poly A Poly T Design-2 4 nt (1 Bulge)
Concentration (nM)
Time (hrs)
Input duplex
20 nM
50 nM
100 nM
200 nM
500 nM
1000 nM

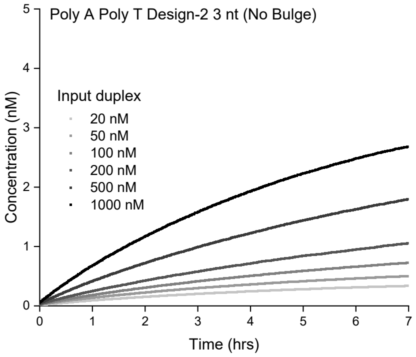

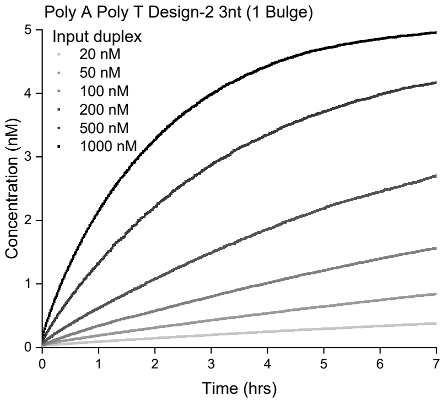

Figure S2. Extensive Characterisation of Reaction Kinetics for 1 bulge system (Poly A/Poly T, Toehold Design-2). We have tested 24 different conditions (6 concentrations and 3-6 nt toeholds) to quantify how the presence of a bulge accelerates strand exchange reactions. Acceleration was observed under all 24 conditions tested.. The 3 nt toehold reactions in the absence of a bulge were too slow to be characterised in Figure 4c. X axis is 15 minutes for 5, 6 nt toehold reactions and 7 hours for 3, 4, nt toehold reactions.

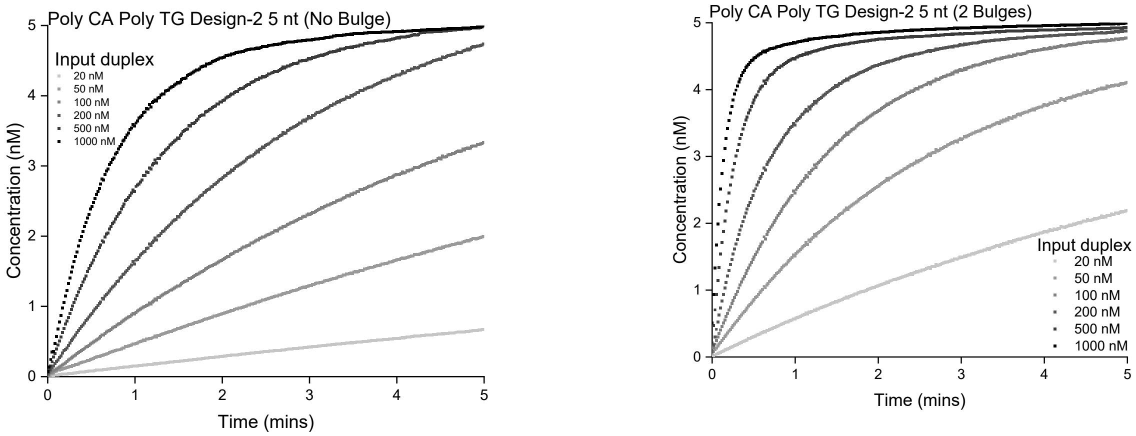

Figure S3. Reaction Kinetics for Poly CA Poly TG Design-2 with 5 nt toeholds in the presence or absence of bulges. Similar to Figure 3b and c, presence of bulges accelerated the strand exchange reactions. The overall speed is somewhat slower than 3b and c but still comparable.

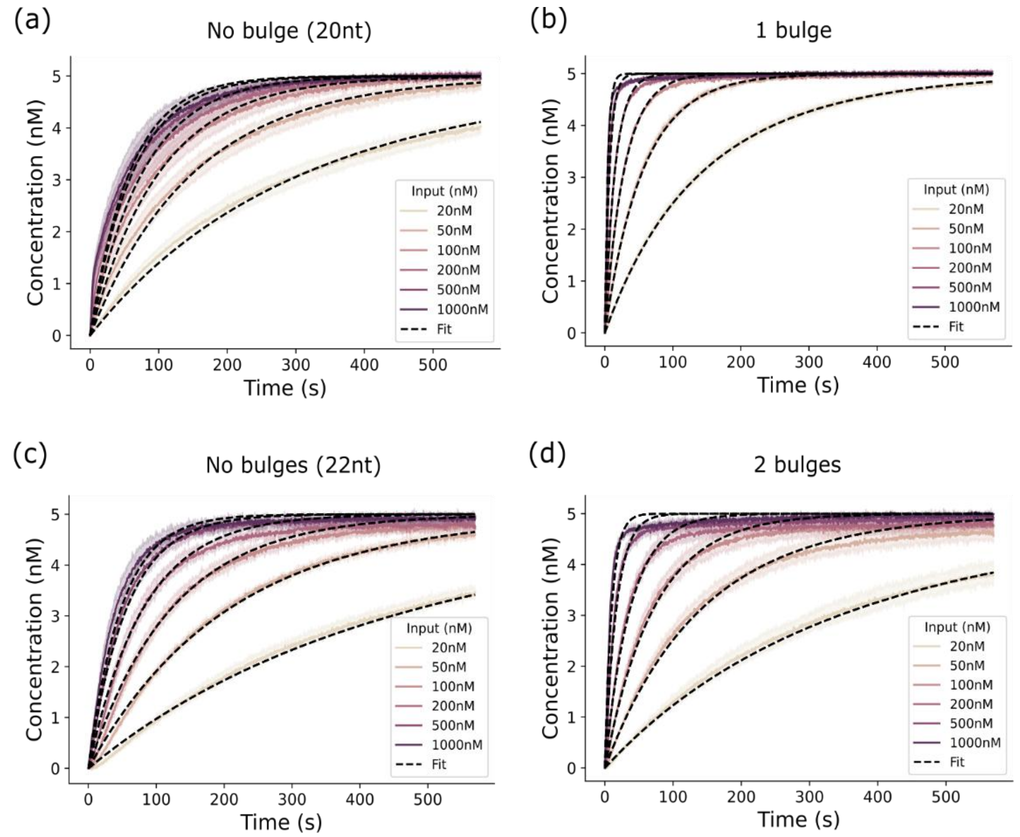

Figure S4. Fits to fluorescence traces under the assumption of $k_{on} = 10^8 M^{-1}\ s^{-1}$. Normalised fluorescent traces taken from Figures 2c–d and 3b–c where 5 nM of probe duplex were mixed 20 nM – 1000 nM of input duplex in the absence (a) or presence (b) of 1 bulge and the absence (c) or presence (d) of two bulges. Black, dashed lines represent fits to the experimental data.

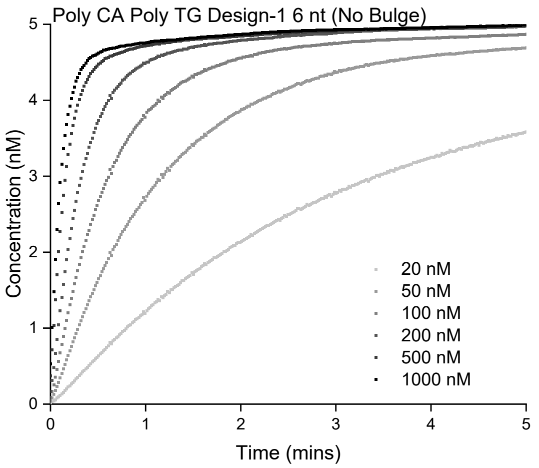
Poly CA Poly TG Design-1 6 nt (No Bulge)
Concentration (nM)
Time (mins)
20 nM
50 nM
100 nM
200 nM
500 nM
1000 nM

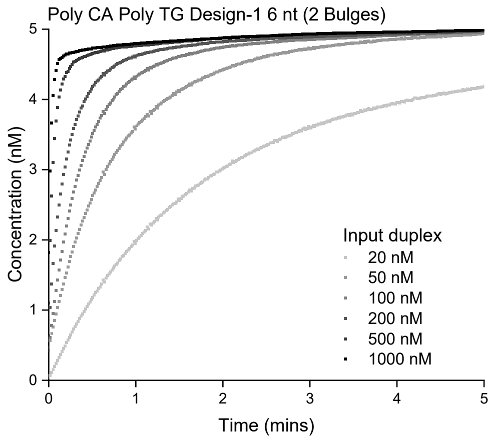
Poly CA Poly TG Design-1 6 nt (2 Bulges)
Concentration (nM)
Time (mins)
Input duplex
20 nM
50 nM
100 nM
200 nM
500 nM
1000 nM

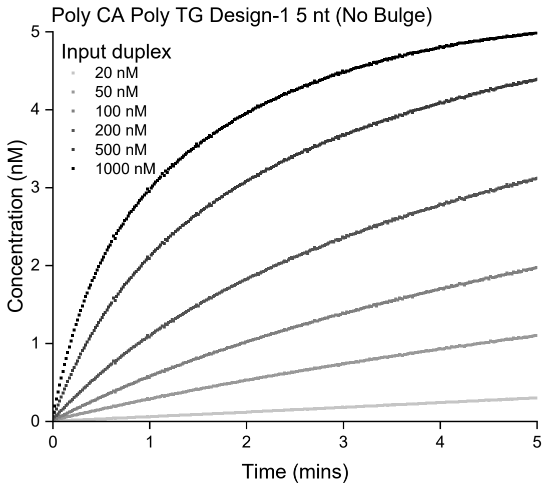
Poly CA Poly TG Design-1 5 nt (No Bulge)
Concentration (nM)
Time (mins)
Input duplex
20 nM
50 nM
100 nM
200 nM
500 nM
1000 nM

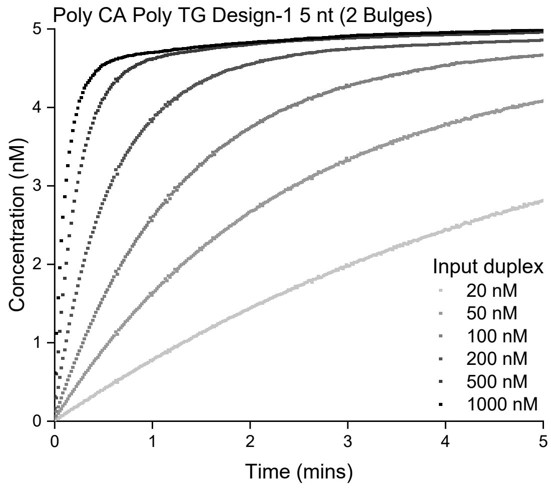
Poly CA Poly TG Design-1 5 nt (2 Bulges)
Concentration (nM)
Time (mins)
Input duplex
20 nM
50 nM
100 nM
200 nM
500 nM
1000 nM

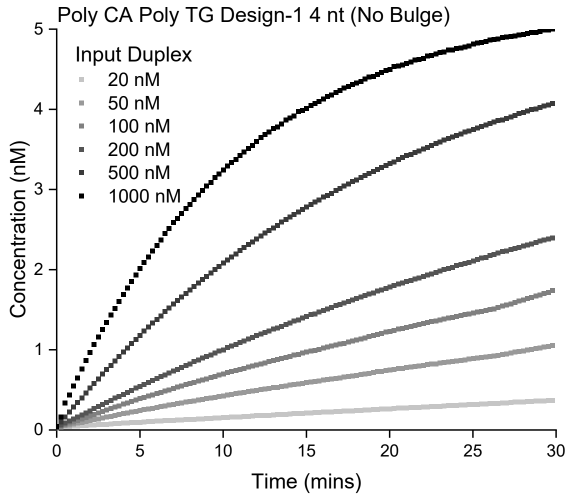
Poly CA Poly TG Design-1 4 nt (No Bulge)
Concentration (nM)
Time (mins)
Input Duplex
20 nM
50 nM
100 nM
200 nM
500 nM
1000 nM

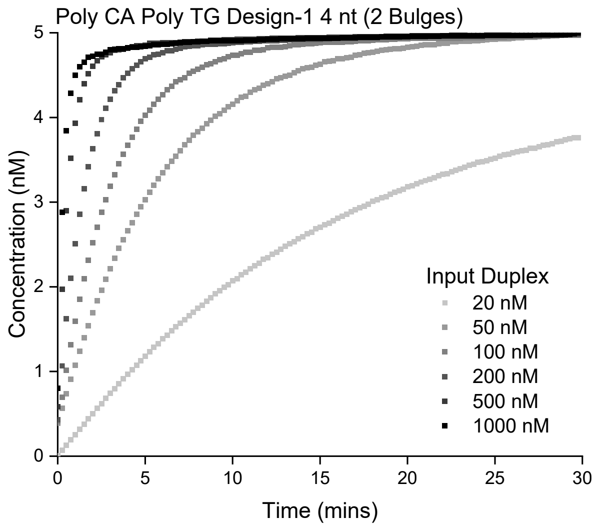
Poly CA Poly TG Design-1 4 nt (2 Bulges)
Concentration (nM)
Time (mins)
Input Duplex
20 nM
50 nM
100 nM
200 nM
500 nM
1000 nM

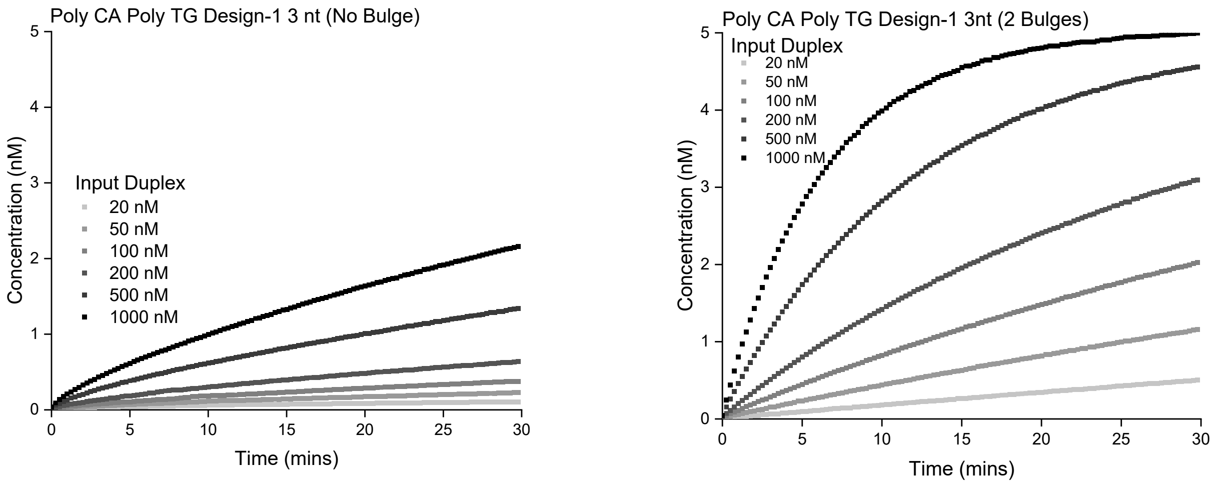

Figure S5. Extensive Characterisation of Reaction Kinetics for 2 bulges system (Poly CA/Poly TG, Toehold Design-1). We have tested 24 different conditions (6 concentrations and 3-6 nt toeholds) to quantify how the presence of bulges accelerates strand exchange reactions. The presence of bulges accelerated the strand exchange reactions in all 24 conditions. The 3 nt and 4 nt toehold reactions in the absence of bulges were too slow to be characterised in Figure 4c.

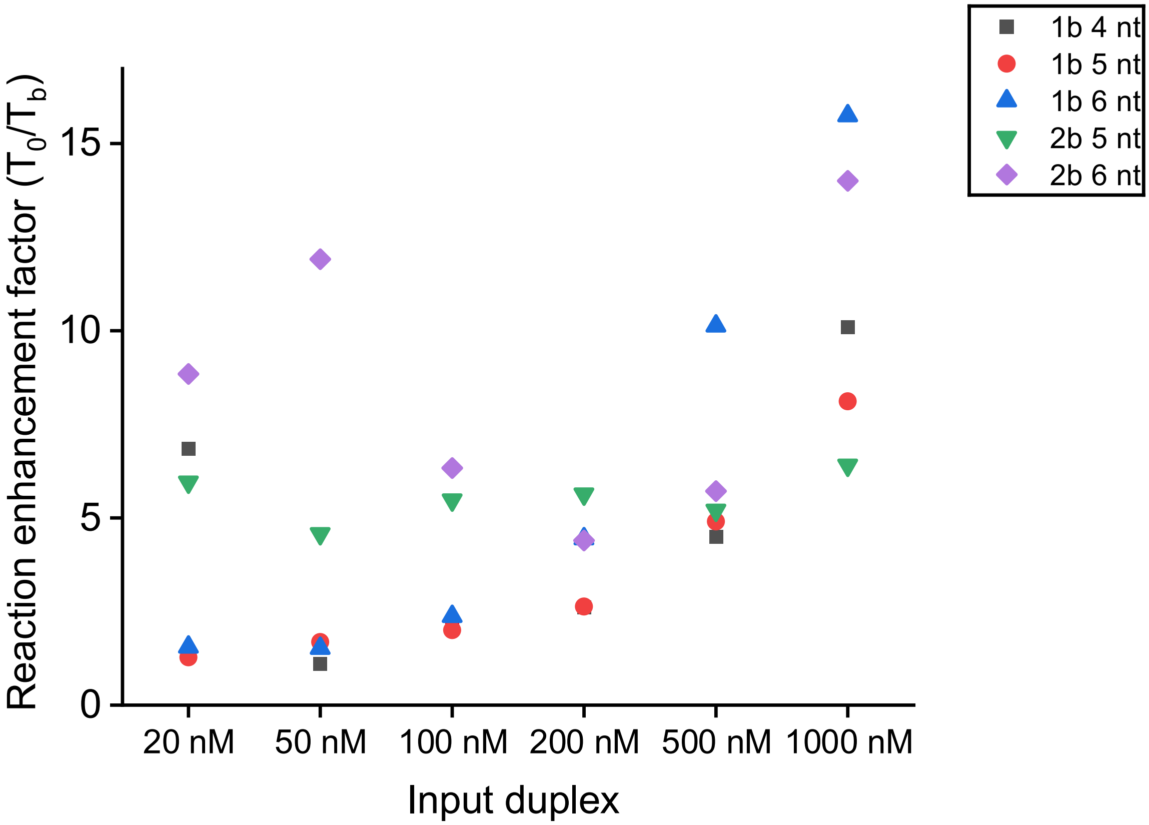

Figure S6. Scatter plot of the Figure 4c: reaction enhancement factor (T0/Tb) for different lengths of toeholds and concentrations of input duplexes.

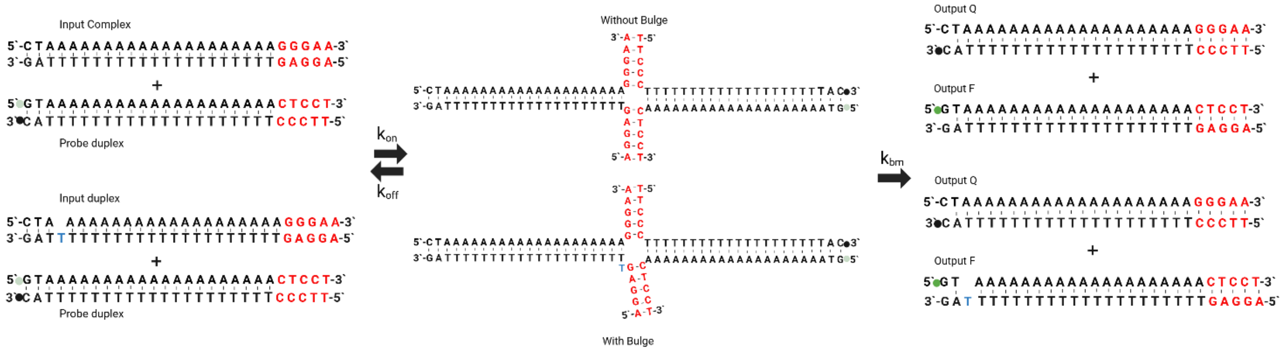

Figure S7. Sequence Schematic for with and without 1-bulge complexes.

Supplementary references
